# Genetic diversity of clinical and environmental Mucorales isolates obtained from an investigation of mucormycosis cases among solid organ transplant recipients

**DOI:** 10.1099/mgen.0.000473

**Published:** 2020-11-27

**Authors:** M. Hong Nguyen, Drishti Kaul, Carlene Muto, Shaoji J. Cheng, R. Alex Richter, Vincent M. Bruno, Guojun Liu, Sinem Beyhan, Alexander J. Sundermann, Stephanie Mounaud, A. William Pasculle, William C. Nierman, Eileen Driscoll, Richard Cumbie, Cornelius J. Clancy, Christopher L. Dupont

**Affiliations:** ^1^​ University of Pittsburgh School of Medicine, Pittsburgh, PA, USA; ^2^​ University of Pittsburgh Medical Center, Pittsburgh, PA, USA; ^3^​ J. Craig Venter Institute, La Jolla, CA, USA; ^4^​ University of Maryland School of Medicine, Baltimore, MD, USA; ^5^​ University of Pittsburgh Graduate School of Public Health, Pittsburgh, PA, USA; ^6^​ J. Craig Venter Institute, Rockville, MD, USA; ^§^​ Present address: Department of Medicine, University of Virginia, Charlottesville, VA, USA

**Keywords:** mucormycosis, whole genome sequence, Mucorales, phylogenetics

## Abstract

Mucormycoses are invasive infections by *Rhizopus* species and other Mucorales. Over 10 months, four solid organ transplant (SOT) recipients at our centre developed mucormycosis due to *Rhizopus microsporus* (*n*=2), *R. arrhizus* (*n*=1) or *Lichtheimia corymbifera* (*n*=1), at a median 31.5 days (range: 13–34) post-admission. We performed whole genome sequencing (WGS) on 72 Mucorales isolates (45 *R*. *arrhizus*, 19 *R*. *delemar*, six *R*. *microsporus*, two *Lichtheimia* species) from these patients, from five patients with community-acquired mucormycosis, and from hospital and regional environments. Isolates were compared by core protein phylogeny and global genomic features, including genome size, guanine–cytosine percentages, shared protein families and paralogue expansions. Patient isolates fell into six core phylogenetic lineages (clades). Phylogenetic and genomic similarities of *R. microsporus* isolates recovered 7 months apart from two SOT recipients in adjoining hospitals suggested a potential common source exposure. However, isolates from other patients and environmental sites had unique genomes. Many isolates that were indistinguishable by core phylogeny were distinct by one or more global genomic comparisons. Certain clades were recovered throughout the study period, whereas others were found at particular time points. In conclusion, mucormycosis cases could not be genetically linked to a definitive environmental source. Comprehensive genomic analyses eliminated false associations between Mucorales isolates that would have been assigned using core phylogenetic or less extensive genomic comparisons. The genomic diversity of Mucorales mandates that multiple isolates from individual patients and environmental sites undergo WGS during epidemiological investigations. However, exhaustive surveillance of fungal populations in a hospital and surrounding community is probably infeasible.

## Data Summary

Genome sequences have been deposited at NCBI under bioproject PRJNA475137: *Rhizopus oryzae* Genome sequencing and assembly. Addtionally, ITS and D1/D2 sequences have been deposited at NCBI under accession numbers MT590526–MT590597 and MT590425–MT590496 respectively. All phylogeny and sequence alignment files have been deposited and published at figshare under https://doi.org/10.6084/m9.figshare.12477767.v1.

**Impact Statement**

Mucormycoses, invasive infections by *Rhizopus* and other Mucorales fungi*,* cause high mortality in immunosuppressed humans. We performed whole genome sequencing (WGS) on 72 Mucorales isolates (45 *Rhizopus arrhizus*, 19 *R*. *delemar*, six *R*. *microsporus*, two *Lichtheimia* species) from four solid organ transplant (SOT) recipients diagnosed with mucormycosis while inpatients, three outpatients at our centre, two patients at other hospitals in the greater Pittsburgh region, and hospital and regional environments. We also included a clinical isolate from a commercial laboratory. This is the first WGS investigation of epidemiologically linked clinical and environmental *Rhizopus* or Mucorales isolates. Mucormycosis cases could not be linked genetically to an environmental source. However, two SOT recipients were infected with highly genetically similar *R. microsporus* isolates that were propably derived from a common parent strain. The study is important for describing the remarkable genetic diversity of disease-causing, hospital environmental and regional *Mucorales* isolates, and for demonstrating that comprehensive core phylogenetic and global genomic analyses are needed to identify isolates as unique and avoid false epidemiological associations. Our comprehensive strain typing strategy coupled with rigorous epidemiologic data provides a model for mucormycosis investigations. This study highlights the strengths and limitations of WGS as a tool for studying mucormycosis.

## Introduction

Mucormycoses are invasive infections caused by fungi of the order Mucorales*,* among which *Rhizopus* species are most common. Mucorales are distributed widely in environmental reservoirs such as soil, vegetation and compost [1]. Mucormycosis usually occurs sporadically among immunosuppressed hosts in healthcare settings or the community, and the disease results in high mortality and morbidity [2, 3]. Healthcare-associated mucormycosis has been ascribed to on-campus construction, as well as contaminated hospital linen, adhesive wrappings, bandages, wooden tongue depressors, drugs, food, ostomy bags and air handling systems [1, 4–19]. The distinction between healthcare-associated and community-acquired mucormycosis often is unclear because the incubation period is unknown, long-term colonization can occur among at-risk patients, and nosocomial case clusters may be caused by multiple species of Mucorales [20].

Until recently, putative healthcare-associated mucormycosis cases were investigated by genotyping of epidemiologically linked clinical and environmental isolates using the internally transcribed spacer (ITS [21]) and D1/D2 regions of the 28S rRNA subunit, or multilocus sequencing typing of conserved loci [22]. Emerging data suggest that these approaches do not provide necessary resolution for discriminating between strains of a particular species, or adequately reflect genome-scale differences in phylogeny [23, 24]. Phylogenetic analysis of whole genome sequence (WGS) data has been applied infrequently in studies of mucormycosis [24–26]. A major challenge for genomic epidemiological studies is that Mucorales phylogeny is poorly understood. The unsettled taxonomy of Mucorales also creates confusion [27, 28]. Recent studies have used WGS to establish preliminary phylogenomic relationships among Mucorales [23, 28], but significant gaps remain in understanding basic genome structures, differences between and within genera and species, and markers of isolate relatedness [28–31]. Previous WGS-based epidemiological studies were limited further by a failure to recover Mucorales during environmental surveillance for comparison with clinical isolates [24, 25]. The genomic variability of Mucorales within hospitals and surrounding communities is unknown [20].

Over a 10-month period, four solid organ transplant (SOT) recipients with mucormycosis were identified in two buildings at hospitals in our medical centre. Three patients were diagnosed in the first 4 months, which raised concerns for a common healthcare source exposure. In this study, we performed WGS and phylogenetic and global genomic analyses on Mucorales isolates from our four patients, and on isolates of the same genera (*Rhizopus* and *Lichtheimia*) that were recovered during environmental surveillance. For reference, we included clinical and environmental Mucorales isolates from the geographical region that were not linked epidemiologically to our hospitalized patients, and a clinical isolate from a repository in Texas. Our primary objective was to determine if mucormycosis cases could be molecularly linked to environmental sources in the hospitals or surrounding community. Secondary objectives were to determine phylogenetic relationships among isolates at our centre and those from elsewhere in the region, temporal changes in Mucorales populations, and genetic diversity within and between different species. A detailed description of the epidemiological investigation of cases will be presented in a separate paper.

## Methods

### Isolate collection

Patient isolates were obtained from the University of Pittsburgh Medical Center Clinical Microbiology Laboratory and the Fungus Testing Laboratory (San Antonio, TX, USA). Environmental culturing was performed using our established methodology [32]. Fungi were identified using lactophenol aniline blue-stained preparations of colonies and by ITS and D1/D2 sequencing. *Rhizopus* isolates were confirmed by the Fungus Testing Lab using standard phenotypic and genotypic methods. Seventy-six isolates underwent WGS. Four isolates (two control clinical isolates from TX, a linen-associated and a regional isolate) were not included in phylogenetic analysis because of poor quality raw data and/or sample redundancy.

### DNA extraction and whole-genome sequencing

Single spores were collected from Mucorales isolates on potato dextrose agar plates, and incubated overnight at 35 °C in 100 ml minimal medium with shaking. Approximately 2.0 g of washed mycelia was frozen with liquid nitrogen. DNA was extracted from ground mycelia with phenol/chloroform [33], and purified with FastDNA spin kit (MP Bio). Illumina libraries were prepared using the Nextera DNA Sample Preparation Kit (Illumina) [34]. After PCR amplification, libraries were cleaned with Ampure XP Reagent (Beckman Coulter), followed by bulk library quantification and normalization by quantitative PCR (qPCR). Libraries were then sequenced with a 2×150-bp paired-ended reads protocol on the Illumina NextSEQ 500 platform, resulting in an average of approximately 13–15 million reads per isolate, across all samples.

### Genome assembly and quality assessment

Reads were demultiplexed according to barcodes followed by quality filtering, and assemblies were generated using SPAdes (v3.8.0) with the following k-mer lengths: 27, 33, 55 and 75 [35]. Following genome assembly, the completeness of all isolate genomes was quantitatively assessed with the Benchmarking Universal Single‐Copy Orthologs (BUSCO) toolkit [36] using lineage-specific orthologues. Each isolate genome was checked for the copy number of 290 BUSCO orthologous groups specific to the fungi_odb9 lineage using the Augustus [37] species ‘*Rhizopus oryzae*’ for genome mode assessment. BUSCO groups were selected from single-copy orthologues that were present in at least 90 % of the species in the OrthoDB v9 database. Note that the orthologous group constructed from BUSCO gene ‘BUSCOfEOG092C2GWG’ was missing from all isolates.

### Gene calling, annotation and phylogenetic inference

Protein-coding genes were predicted with an evidence-based annotation workflow using AUGUSTUS (v3.2.3) [37], from the MAKER suite of tools [38]. We utilized the ‘complete’ mode for the *R. oryzae* (‘rhizopus_oryzae’) gene model, predicting only complete genes for all isolate genomes. Protein sequences and nucleotide coding sequences were then generated from the AUGUSTUS output using getAnnoFasta.pl (available in the AUGUSTUS suite of scripts). The PhyloSift [39] eukaryotic reference marker set was downloaded in the form of sequence alignments and a profile hidden Markov model (HMM) was generated for each eukaryotic marker, which were then concatenated into one combined.hmm for query. The concatenated HMM containing all marker genes was then searched in each of the genomes using hmmsearch and a consolidated table containing the following fields was generated: query gene_id (from AUGUSTUS output), genome_ID, marker_ID (from phylosift marker set) and the gene_sequence with the top domain score, using a minimum e-value threshold of 1e-5. Coding sequences for these genes were then extracted and renamed by genome, and aligned using Clustal Omega v1.2.1 [40]. Alignments were concatenated to make a combined multi-fasta alignment file. To facilitate tree building, the number of markers was filtered down from a set of 33 reference marker genes originally identified to be conserved among all eukaryotes to 15 that were present across all isolate genomes. These were identified as 40S, Actin_noOuts, Atub_noOuts, ef1aLike, ef2_noOuts, enolase, grc5, hsp70cyt, Hsp90, metk_noOuts, Rad51_noOuts, rps22, Rps23a_noOuts, TFIIH and Tsec61. Phylogenetic analysis was performed with RAxML 8.1.20 [41, 42] using the multi-fasta alignments under the following parameters: GTRGAMMA nucleotide substitution model, iterating over 100 bootstraps with an initial seed of 100, and visualized using FigTree v1.4.3 (http://tree.bio.ed.ac.uk/software/figtree/).

### Clade-specific pangenome analysis

Genomes were organized into clades. In order to identify a core genome within a clade, we employed Pangenome Ortholog Clustering Tool (PanOCT) [43] to predict orthologous clusters in these ‘pan-genomes’ by utilizing all-vs-all blast results, conservation of gene order and orientation within the genomes of closely related species (CGN) to discrimate between orthologues and paralogues. blastn was used for the all-vs-all search in each genome, using the run_pangenome.pl script. GenBank files were generated from GFF and sequence fasta files for each genome using gff2gbSmallDNA.pl in the AUGUSTUS suite of scripts. The pipeline generated several output files, the most informative of which were match_table* files. These files contained orthologue cluster information, followed by subsequent genomes as additional columns. The columns were ordered according to the list of genomes given as input, listing the percentage identity of each representative protein in that genome corresponding to the reference, and the core_cluster* file. Orthologous clusters were classified into core (clusters that have a representative in every genome), shared (clusters that are present in two or more genomes), and unique or singletons (genes that are unique to a single genome). PanOCT was run using default values for blast E-value cut-off (1e^−5^), sequence identity threshold (35%) and minimum match length cut-off.

## Results

### Mucorales clinical and environmental isolates

**Fig. 1.**
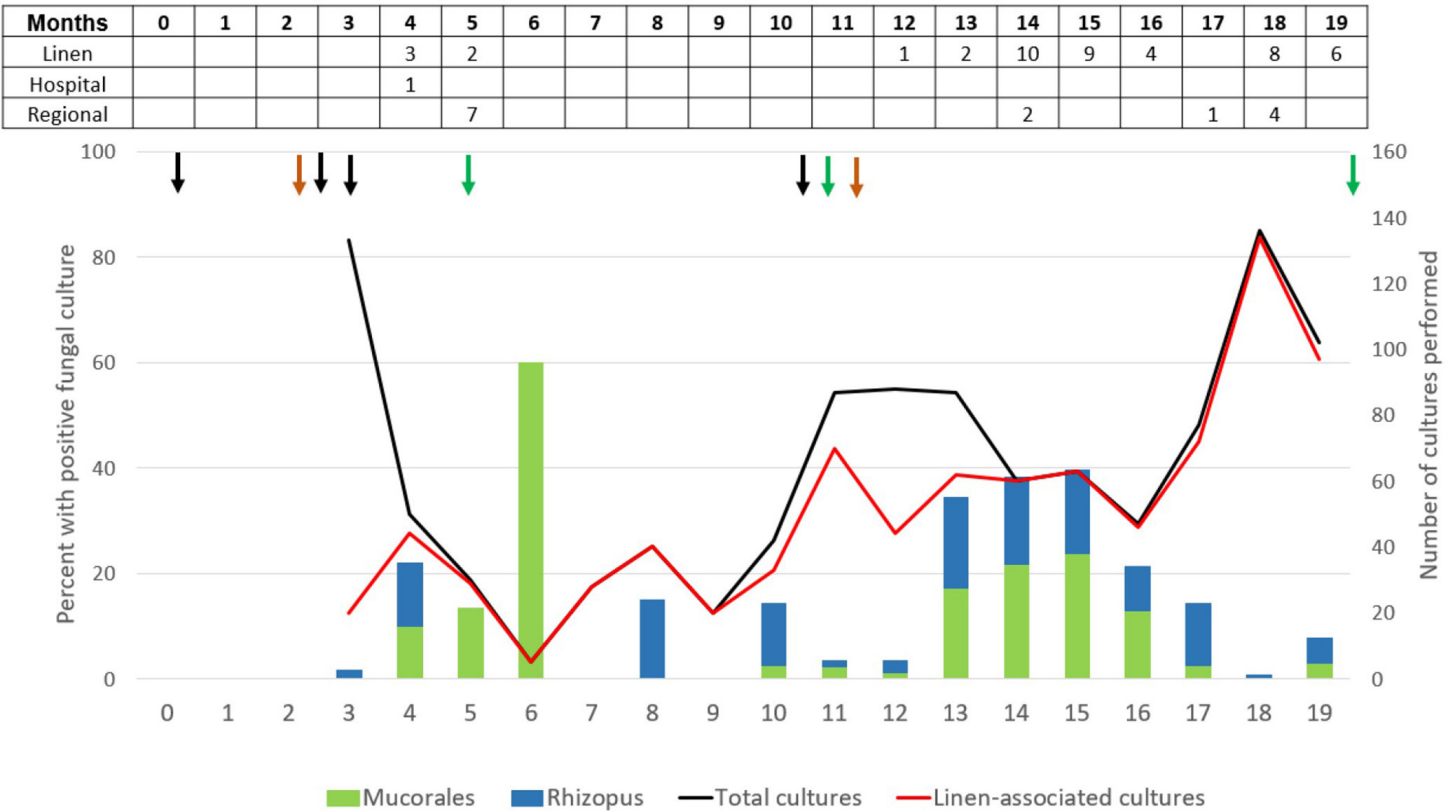
Timeline of Mucorales recovery from patients and environmental cultures. Bar graphs represent the percentages of environmental cultures that were positive in a given month for *Rhizopus* (blue bars) and other Mucorales species (green bars). Line graphs represent the total number of environmental cultures performed in a month (black line; includes environmental cultures at our hospitals, cultures of freshly laundered linen and linen carts immediately upon delivery to our centre, and cultures at an outside linen agency) and linen-associated cultures only (red line; includes freshly laundered linen and linen carts immediately upon delivery to our centre, and cultures at the outside linen agency). The inset Table presents the number of *Rhizopus*/*Lichtheimia* isolates that underwent WGS. As described in the text, isolates are classified as linen-associated, hospital environment or regional environmental isolates. Arrows beneath the inset Table signify patient isolates. Black arrows denote isolates from patients with mucormycosis diagnosed while inpatients at our hospitals. Green arrows denote isolates with community-acquired mucormycosis. Orange arrows denote isolates from patients admitted to outside hospitals. One patient isolate obtained from the Fungus Testing Lab in San Antonio, TX, which served as a control, is not shown.

The timeline of Mucorales recovery from patient cultures and environmental cultures is presented in Fig. 1. Between study months 0 and 10, four SOT recipients developed mucormycosis at a median of 31.5 days (range: 13–34) following hospital admission. Each patient was cared for exclusively in one of two buildings at hospitals in our medical centre, which are separated by a city block and connected by a single floor walkway that traverses an intervening building. New and freshly laundered linen was provided to both hospitals from an offsite agency. Three SOT recipients in hospital buildings A (*n*=2) and B (*n*=1) were diagnosed in months 0–4 with infections by *Rhizopus arrhizus var*. *delemar* (*R. delemar*)*, Rhizopus microsporus* and *Lichtheimia corymbifera*, respectively, as identified based on standard morphological characteristics and later genotypically identified using ITS and D1/D2 sequencing. In month 10, a fourth SOT recipient was diagnosed with an *R. microsporus* infection in hospital building B. Between months 5 and 19, three other patients presented to hospital building A or B with community-acquired mucormycosis (i.e. no prior hospital contact) due to *Rhizopus arrhizus var. arrhizus* (*R. arrhizus*).

Between months 3 and 19, *Rhizopus* and *Lichtheimia* isolates were recovered from surveillance cultures of freshly laundered linen or carts containing these linen items immediately upon arrival at our medical centre, laundered linen or environmental sites at the offsite agency, and the environment in our medical centre (Fig. 1).

### WGS of *Rhizopus*/*Lichtheimia* isolates

Seventy-two isolates that underwent WGS (Illumina NextSeq) were included in the study (Table S1, Fig. S1, available in the online version of this article). By ITS and D1/D2 sequencing, isolates were identified as *R. arrhizus* (*n*=45), *R. delemar* (*n*=19), *R. microsporus* (*n*=6) and *Lichtheimia* spcies (*n*=2). Isolates were classified into four groups.

#### Patient isolates

Isolates in this group were designated as P (patient), followed by the isolate number. The group included 12 isolates from 10 patients. For one patient, colonies derived from the same parent strain were sequenced independently (P6-GL35 and P6-GL58). In a second patient, a pair of longitudinal isolates were sequenced (P7-GL36 and P7-GL60). Overall, five isolates were sequenced from our four SOT recipients who were diagnosed with mucormycosis while inpatients (P1, P3, P4, P6); four isolates were sequenced from three patients (two SOT recipients) who were admitted to our hospitals with community-acquired mucormycosis (P5, P9 and P7). For external reference, we included isolates from two patients with mucormycosis diagnosed at other hospitals in the greater Pittsburgh region (P2 and P8), and a clinical isolate obtained from the Fungus Testing Laboratory in San Antonio, TX (P10-GL56).

#### Linen-associated isolates

These isolates (*n*=45) were designated as L, followed by isolate number. The group included 27 isolates recovered directly from linen or linen carts immediately upon arrival at our medical centre, and 18 isolates from laundered linen, air and environmental surfaces at the offsite linen agency.

#### Regional environmental isolates

Isolates were designated as R, followed by isolate number. The group included 14 isolates recovered from extra-hospital environmental sites in the Pittsburgh region.

#### Hospital environment isolate

A *Lichtheimia hongkongensis* isolate (N10) was recovered in month 4 after deconstructing the walls of an intensive care unit (ICU) in hospital building A that housed SOT recipients.

### Phylogenetic analysis using ITS and D1/D2 sequencing and WGS

Phylogenetic relationships between *Rhizopus* isolates were assessed initially using ITS and D1/D2 sequencing and phylogenetic inference. ITS sequences did not resolve fine-scale relationships between the isolates, instead collapsing them into two distinct phylogenetic clades without internal clade support (Fig. S1). Phylogenetic inferences based on D1/D2 recapitulated this bifurcation. While the two D1/D2 clades had intraclade topology showing variations in the D1/D2 region, these were not supported by bootstrap values.

**Fig. 2.**
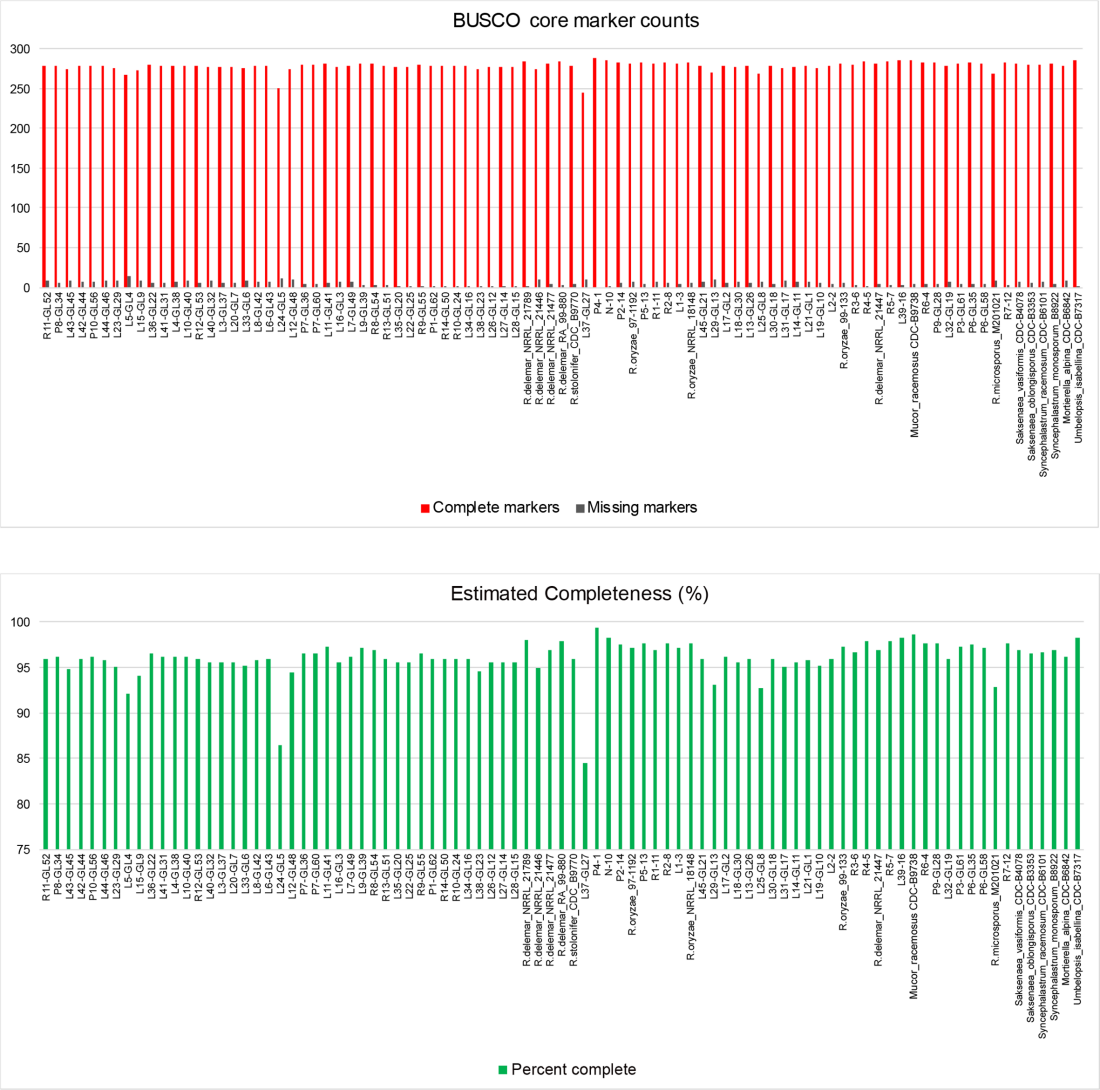
Assessment of genome completeness using BUSCOs. Analyses were for near universal single copy orthologues selected from fungal sets (OrthoDB v9). Note that the genomes of all except two of the newly sequenced isolates and existing reference strains achieved a near universal level of completion based on the 290 BUSCO core marker genes.

We employed WGS to further investigate relationships among *Rhizopus* and *Lichtheimia* isolates. Approximately 3.5 Gbp was obtained for each genome and assembled using SPAdes [44]. Assembly statistics are provided in Table S1. For reference, we included 17 previously sequenced Mucorales strains [28]. The assemblies were fragmented, which is typical for the repeat-rich *Rhizopus* genomes. Despite this fragmentation, the newly sequenced and existing reference genomes had a near universal level of completion based on 290 BUSCO (Benchmarking Universal Single‐Copy Orthologs) core marker genes [36]. L24-GL5 and L37-GL27 were the only isolates that were less than 90 % complete (Fig. 2).

Phylogenetic inferences based on concatenated sequences of 15 conserved marker proteins provided clarity on the number of distinct lineages that were isolated. Low bootstrap values and nearly identical core protein alignments of internal groupings prevented precise information about fine-scale phylogenetic relatedness. Fortunately, the high level of genome completeness across the dataset facilitated comparative analyses of genome size, guanine–cytosine percentage (%GC), and core- and pan-genomes. Core- and pan-genome analyses were performed in two ways. First, proteins were clustered into families. When protein family content between a subset of genomes did not provide resolution, the PanOCT was used to identify genome-specific paralogue expansions based on gene neighbourhood conservation [43]. These analyses allowed us to infer phylogenetic relationships based on anticipated protein content in addition to evolutionary history.

### Phylogenomic relatedness of Mucorales isolates

**Fig. 3.**
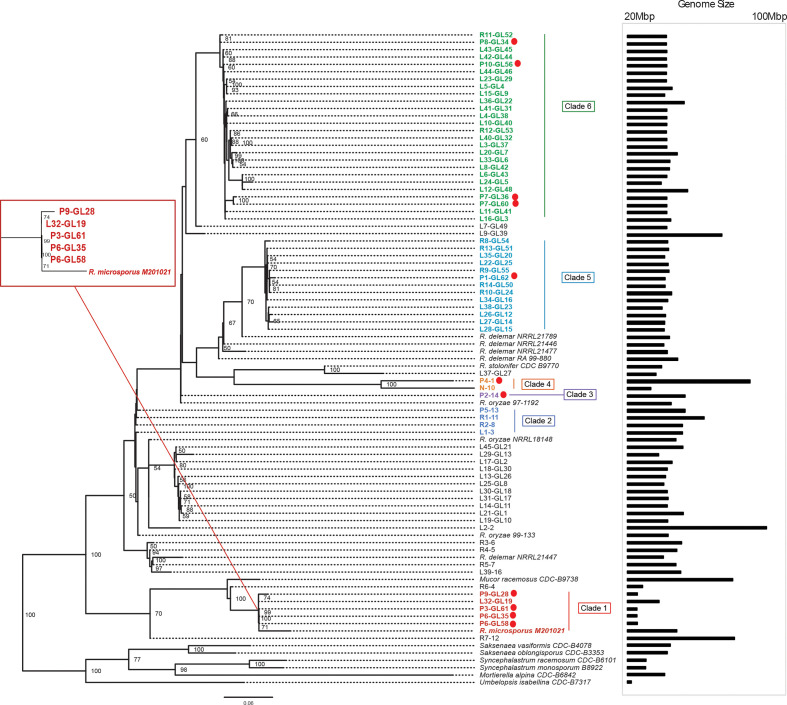
Phylogenetic relatedness of Mucorales isolates, based on concatenated sequences of conserved core marker proteins. Patient isolates (labelled as ‘P’ followed by isolate number, and indicated in the figure by red dots) fell into six clades (clades I–VI). Linen-associated (labelled as ‘L’ followed by isolate number) and regional environment isolates (labelled as ‘R’ followed by isolate number) are also shown, as are reference genomes from a previous study [27]. The bar chart to the right of the figure shows genome sizes for the isolates. Bootstrap values above 50 are shown. The phylogenetic relationship of Clade 1 is magnified and shown in the inset on the left. The scale bar represents the number of substitutions per site (0.06).

**Fig. 4.**
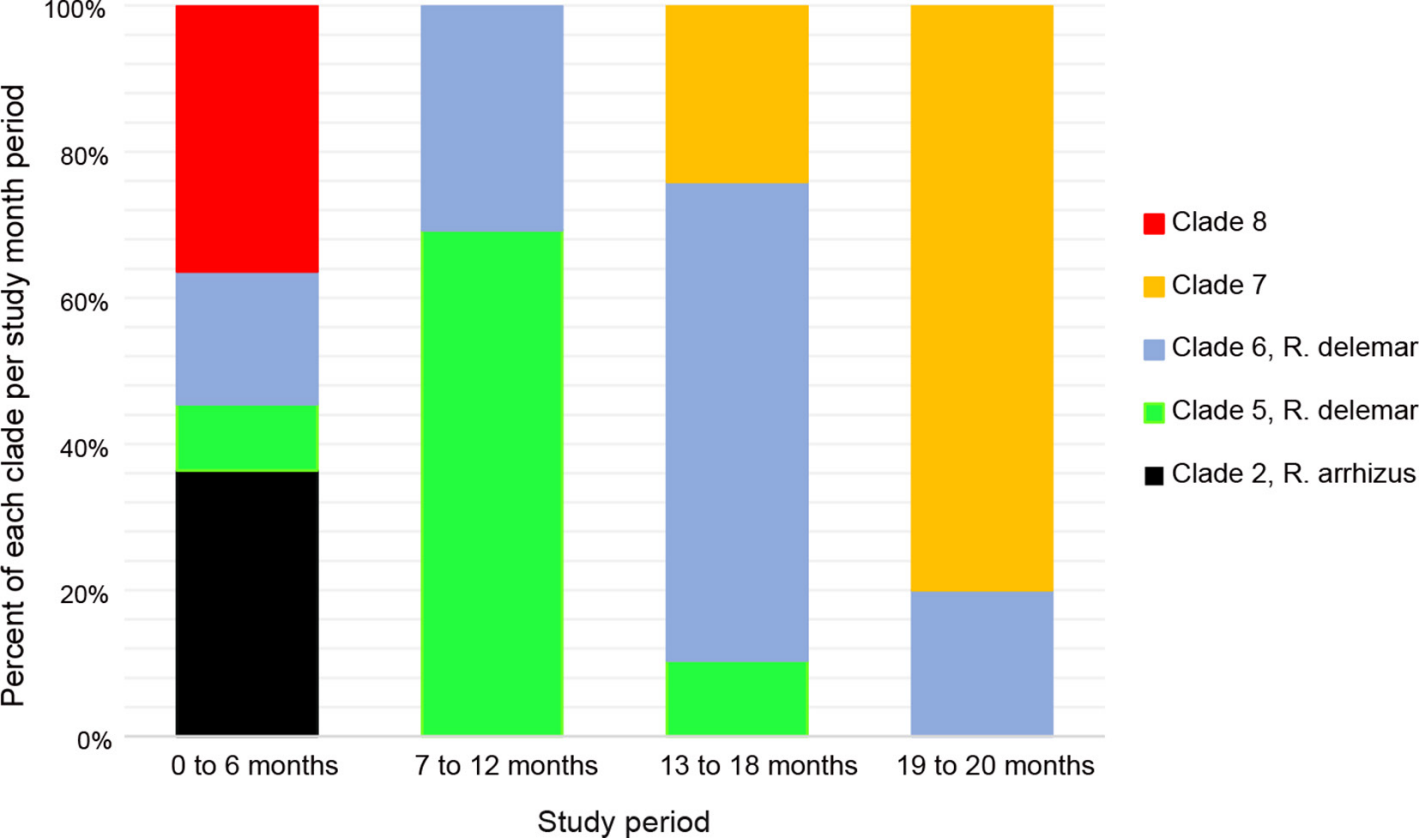
Recovery of *R. arrhizus* and *R. delemar* clades over time.

Using data from the core protein phylogenetic analysis, we assigned patient isolates into six phylogenetic lineages (clades 1–6) (Fig. 3). Two other clades (7 and 8) included linen-associated and regional isolates but not patient isolates. Descriptions and timelines of recovery of isolates in each clade are summarized in Fig. 4 and Table 1. Clade-specific results are discussed in detail below.

**Table 1.** Mucorales isolates that underwent WGS, organized by core protein phylogeny

Clade types of isolates	Isolate	Species identification by ITS and D1/D2	Time frame of recovery (study month)	Description of patient isolates
**Clade 1**				
Reference: *R. microsporus* M201021				
Patient isolates	P3-GL61	*R. microsporus*	3	Isolate associated with mucormycosis diagnosed while inpatient (Hospital A)
	P6-GL35	*R. microsporus*	11	Isolate associated with mucormycosis diagnosed while inpatient (Hospital B)
	P6-GL58	*R. microsporus*	11	Isolate from same parent strain as P6-GL35, independently sequenced
	P9-GL28	*R. microsporus*	19	Isolate associated with community-acquired mucormycosis
Linen-associated isolate	L32-GL19	*R. microsporus*	18	
Regional environmental isolate	R6-4	*R. microsporus*	5	
**Clade 2**				
Reference: *R. oryzae* 99-133				
Patient isolate	P5-13	*R. arrhizus var. arrhizus*	5	Isolate associated with community-acquired mucormycosis
Linen-associated isolate	L1-3	*R. arrhizus var. arrhizus*	5	
Regional environmental isolates	R1-11	*R. arrhizus var. arrhizus*	5	
	R2-8	*R. arrhizus var. arrhizus*	5	
**Clade 3**				
Reference: *R. oryzae* 97-1132				
Patient isolate	P2-14	*R. arrhizus var arrhizus*	3	Isolate obtained from a patient at an outside hospital
Linen-associated isolates	None			
Regional environmental isolates	None			
**Clade 4**				
Reference: *R. stolonifer* CDC-B9770				
Patient isolate	P4-1	*Lichtheimia corymbifera*	4	Isolate associated with mucormycosis diagnosed while inpatient (Hospital B)
Linen-associated isolates	None			
Hospital environment isolate	N-10	*Lichtheimia hongkongensis*	5	Environmental isolate within a deconstructed dry wall of Hospital A
**Clade 5**				
Reference: *R. delemar* NRRL21789				
Patient isolate	P1-GL62	*Rhizopus arrhizus var. delemar*	0	Isolate associated with mucormycosis diagnosed while inpatient (Hospital A)
Linen-associated isolates	L22-GL25	*Rhizopus arrhizus var. delemar*	16	
	L26-GL12	*Rhizopus arrhizus var. delemar*	18	
	L27-GL14	*Rhizopus arrhizus var. delemar*	18	
	L28-GL15	*Rhizopus arrhizus var. delemar*	18	
	L38-GL23	*Rhizopus arrhizus var. delemar*	20	
	L35-GL20	*Rhizopus arrhizus var arrhizus*	20	
	L34-GL16	*Rhizopus arrhizus var. delemar*	19	
Regional environmental isolates	R8-GL54	*Rhizopus arrhizus var. delemar*	14	
	R9-GL55	*Rhizopus arrhizus var. delemar*	14	
	R10-GL24	*Rhizopus arrhizus var. delemar*	18	
	R13-GL51	*Rhizopus arrhizus var. delemar*	18	
	R14-GL50	*Rhizopus arrhizus var. delemar*	18	
**Clade 6**				
Reference: *R. oryzae* 97-1192				
Patients’ isolates	P7-GL36	*R. arrhizus var. arrhizus*	11	Isolate associated with community-acquired mucormycosis
	P7-GL60	*R. arrhizus var. arrhizus*	11	Same patient (P7), isolated 9 days apart
	P8-GL34	*R. arrhizus var. arrhizus*	11	Isolate obtained from a patient at an outside hospital
	P10-GL56	*R. arrhizus var. arrhizus*	(not known)	Clinical isolate from the Fungus Testing Lab
Linen-associated isolates	L40-GL32	*R. arrhizus var. arrhizus*	4	
	L41-GL31	*R. arrhizus var. arrhizus*	4	
	L3-GL37	*R. arrhizus var. arrhizus*	12	
	L4-GL38	*R. arrhizus var. arrhizus*	13	
	L6-GL43	*R. arrhizus var. arrhizus*	14	
	L10-GL40	*R. arrhizus var. arrhizus*	14	
	L11-GL41	*R. arrhizus var. arrhizus*	14	
	L12-GL48	*R. arrhizus var. arrhizus*	14	
	L5-GL4	*R. arrhizus var. arrhizus*	14	
	L8-GL42	*R. arrhizus var. arrhizus*	14	
	L42-GL44	*R. arrhizus var. arrhizus*	14	
	L43-GL45	*R. arrhizus var. arrhizus*	14	
	L44-GL46	*R. arrhizus var. arrhizus*	14	
	L15-GL9	*R. arrhizus var. arrhizus*	15	
	L16-GL3	*R. arrhizus var. arrhizus*	15	
	L20-GL7	*R. arrhizus var. arrhizus*	15	
	L23-GL29	*R. arrhizus var. arrhizus*	16	
	L24-GL5	*R. arrhizus var. arrhizus*	16	
	L33-GL6	*R. arrhizus var. arrhizus*	18	
	L36-GL22	*R. arrhizus var. arrhizus*	20	
Regional environment isolates	R11-GL52	*R. arrhizus var. arrhizus*	18	
	R12-GL53	*R. arrhizus var. arrhizus*	18	
**Clade 7**				
Reference: *R. oryzae* NRRL18148				
Patient isolates	None			
Linen-associated isolates	L13-GL26	*Rhizopus arrhizus var. arrhizus*	15	
	L14-GL11	*Rhizopus arrhizus var. arrhizus*	15	
	L17-GL2	*Rhizopus arrhizus var. arrhizus*	15	
	L19-GL10	*Rhizopus arrhizus var. arrhizus*	15	
	L18-GL30	*Rhizopus arrhizus var. arrhizus*	15	
	L21-GL1	*Rhizopus arrhizus var. arrhizus*	15	
	L25-GL8	*Rhizopus arrhizus var. arrhizus*	16	
	L31-GL17	*Rhizopus arrhizus var. arrhizus*	18	
	L30-GL18	*Rhizopus arrhizus var. arrhizus*	18	
	L45-GL21	*Rhizopus arrhizus var. arrhizus*	20	
	L29-GL13	*Rhizopus arrhizus var. delemar*	19	
Regional environmental isolates	None			
**Clade 8**				
Patient isolates	None			
Linen-associated isolate	L39-16	*Rhizopus arrhizus var. delemar*	4	
Regional environmental isolates	R3-6	*Rhizopus arrhizus var. delemar*	5	
	R4-5	*Rhizopus arrhizus var. delemar*	5	
	R5-7	*Rhizopus arrhizus var. delemar*	5	
**Singletons**				
Linen-associated isolates	L37-GL27	*R. arrhizus var. delemar*	20	
	L2-2	*Rhizopus arrhizus var. delemar*	5	
	L7-GL49	*R. arrhizus var. arrhizus*	14	
	L9-GL39	*Rhizopus arrhizus vararrhizus*	14	
Regional environmental isolate	R7-12	*Rhizopus arrhizus var. arrhizus*	5	

Clade 1 comprised six isolates identifed as *R. microsporus* based on morphology and ITS and D1/D2 sequencing, including four isolates from three patients (recovered in study months 3–19), a linen-associated isolate (month 18) and a regional environmental isolate (month 5). In the phylogenomic inference, this clade was closest to reference strain *R. microsporus* M201021.

**Fig. 5.**
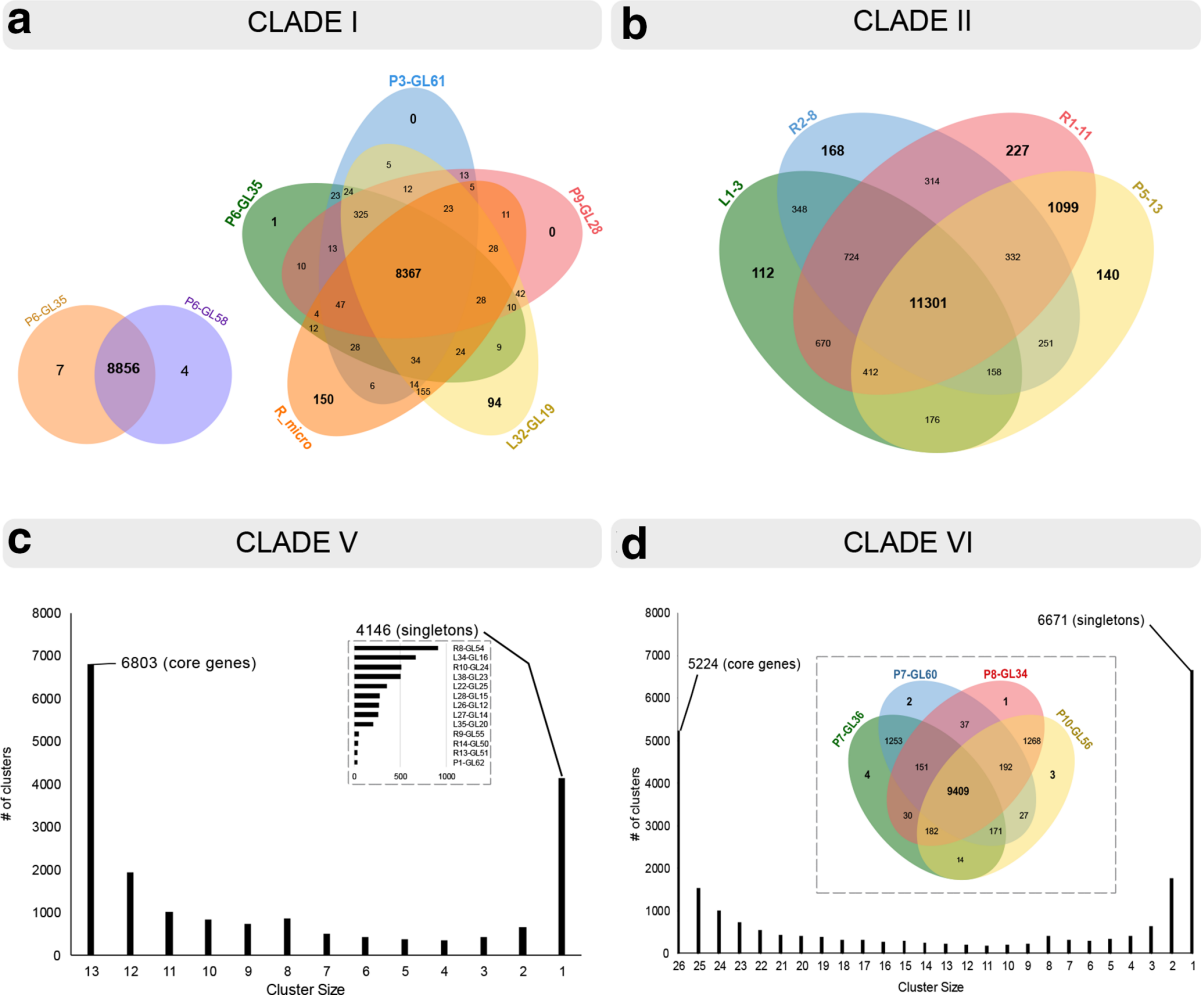
Pan-genome comparisons of protein content for Clades 1, 2, 5 and 6. (a) Five-way Venn diagram comparing Clade 1 *R*. *microsporus* isolates, including three patient isolates (P6-GL35, green; P3-GL61, blue; and P9-GL28, red), a linen-associated isolate (L32-GL19, yellow) and *R. microsporus* reference strain M201021. Each ellipse shows in sum the total number of coding sequences of one strain. Intersections indicate predicted shared content. P3-GL61, P6-GL35 and P9-GL28 were highly similar in pan-genome protein content. However, P9-GL28 differed from the other two isolates by core protein phylogeny (Fig. 2). L32-GL19, on the other hand, was clearly distinct from the patient isolates by pan-genome protein content (as shown here), as well as by genome size and %GC (Fig. 2, Table S1). (b) Four-way Venn diagram comparing the pan-genome protein content comparisons of four Clade 2 *R*. *arrhizus* isolates that were closely related by core protein phylogeny. Included in the diagram are a patient isolate associated with community-acquired mucormycosis (P5-13, yellow), a linen-associated isolate (L1-3, green), and two regional environment isolates (R1-11, red; and R2-8, blue). There was clear divergence in genomes of isolates within this clade. (c) Distribution of protein cluster sizes generated from the comparison of genomes of 13 isolates in Clade 5 using PanOCT. Numbers on the *x*-axis signify the number of isolates that share a given number of genes indicated on the *y*-axis. For example, 6803 protein families were shared by all 13 isolates. The inset bar plot shows distributions for genes that were carried by a single isolate. The genome of each isolate contained a large number of unique protein families. (d) Distribution of protein cluster sizes generated from the comparison of genomes of 26 isolates in Clade 6 using PanOCT. Only 5224 protein families were shared by all isolates. Similar to genomes of Clade 5 isolates, each genome of a Clade 6 isolate contained a large number of unique protein families. The inset figure shows the our-way Venn diagram for the protein content of four patient-derived isolates in Clade 6.

We first performed WGS on two *R*. *microsporus* isolates from patient P6 that were isolated from the same parent strain on potato dextrose agar slants and stored either at −80 °C (P6-GL35) or at room temperature (P6-GL58) for 10 months. The genomes were nearly identical in core protein phylogeny (Fig. 3), genome size (Fig. 3, Table S1), %GC (Table S1) and pan-genome protein content (Fig. 5). For clarity of discussion below, we will use P6-GL35 as the representative isolate from patient P6.

Patient and linen-associated isolates clustered together, and they were distant from regional isolate R6-4, suggesting spatial and possibly temporal diversity (Fig. 3). Isolates from the two SOT inpatients (P6-GL35 and P3-GL61) were distinct from community-acquired isolate P9-GL28 by core protein phylogeny (Fig. 3). These isolates were highly similar to linen-associated isolate L32-GL19 by core protein phylogeny (Fig. 3), but they were clearly distinct by genome size, %GC and protein coding content (Fig. 5a, Table S1). L32-GL19 contained an abundance of protein-coding sequences that were missing from the clinical isolates (Fig. 5a).

In summary, core phylogenetic similarities between patient and linen-associated isolates were obviated by genome-scale comparison. Global genomic similarity between isolates from inpatients P6 and P3 suggested a common source exposure, but the isolates were distinct from any environmental isolate. These patients were housed in different hospitals at our centre and developed mucormycosis 7 months apart (months 3 and 10).

Clade 2 comprised four isolates (community-acquired mucormycosis, linen-associated and two regional isolates) that were recovered in month 5, and identifed as *R. arrhizus* by ITS and D1/D2 sequencing (Table 1, Figs 3 and 5b).

Isolate P5-13 had 98 and 99.9% nucleotide similarity to regional isolates R2-8 and R1-11, respectively, but slightly lower similarity to L1-3 (97 %). Additionally, pairwise comparison of the genomic content among the four isolates revealed >97 % identity. Isolates P5-13, R2-8 and L1-3 differed from R1-11 by having smaller genomes (49 versus 60 Mbp) and fewer total predicted proteins (17 834 versus 21 771 proteins), indicating genomic divergence. The P5-13 genome contained 1822 protein families that were not present in L1-3. Conversely, L1-3 had 1854 protein families that were lacking in P5-13, demonstrating that the genomes were distinct. Similarly, the genomes of P5-13 and R2-8 differed from each other by 3381 protein families.

In summary, there was clear divergence between isolates within this clade. Community-acquired mucormycosis in patient P5 was not caused by an isolate recovered from the environment.

Clade 3 comprised a single *R. arrhizus* isolate (P2-14) recovered from a patient at an outside regional hospital in month 3. P2-14 was not phylogenetically similar to other isolates in the study, which precluded source anchoring (Fig. 3). The unique genome and recovery from another hospital suggest spatial diversity.

Clade 4 comprised two isolates identified as *L. corymbifera* and *L. hongkongensis* by ITS and D1/D2 sequencing (Fig. 3). P4-1 (*L. corymbifera*) was recovered from an SOT inpatient at hospital B in month 4. The phylogenetically closest strain (N-10, *L. hongkongensis*) was isolated in month 5 within a deconstructed ICU dry wall in hospital A. P4-1 had an enlarged genome of 84 Mbp (largest in this study), compared to a 32-Mbp genome for N-10.

Clade 5 comprised 12 isolates identifed as *R. delemar* by ITS and D1/D2 sequencing, including an isolate from index SOT patient P1 (month 0), six linen-associated isolates (months 16–20), and five regional environmental isolates (months 14–18). Isolate L35-GL20, identified by ITS and D1/D2 as *R. arrhizus*, also clustered within this clade.

The P1-GL62 genome was remarkably similar to those of other isolates within this clade based on core protein phylogeny, genome sizes, %GC and predicted proteome sizes (Fig. 3, Table S1). However, there was notable diversity in encoded protein families (Fig. 5c). Only 6803 out of 12000–14000 total predicted proteins were shared, and each genome contained a large number of unique protein families.

In summary, clade 5 isolates were found throughout the geographical region during the entire study period (Fig. 4), but none of them were genetically related. Mucormycosis in P1 was not caused by an isolate recovered from the environment.

Clade 6 comprised 26 isolates identifed as *R. arrhizus* by ITS and D1/D2 sequencing, including four isolates recovered from three patients (P7, P8 and P10) in month 11, 20 linen-associated (months 4–20) isolates and two regional isolates (month 18; Table 1). Community-acquired mucormycosis isolates P7-GL36 and P7-GL60 (recovered from the same patient 9 days apart) were closely related in core protein phylogeny (Fig. 3), and showed virtual identity in genome size, %GC and predicted proteome size (Table S1). Protein family and PanOCT analyses demonstrated that the isolates differed from each other by 488 and 2085 protein families, respectively (Fig. 5d, inset). There was marked diversity in encoded protein families among other isolates in the clade, and between other isolates and P7-GL36 or P7-GL60 (Fig. 5d).

In summary, isolates in this clade were genetically distinct and geographically widespread, and there were no links between environmental isolates and those from individual patients. Isolates were recovered throughout the study period, suggesting persistence within resilient environmental reservoirs (Fig. 4).

#### Remaining isolates

The remaining 20 isolates, none of which were patient-derived, were divided into two clades (15 isolates) and five singletons. Clade 7 comprised 11 isolates, including linen-associated *R. arrhizus* (*n*=10) and *R. delemar* (*n*=1). These isolates were recovered starting in month 15 until the end of the study (Fig. 4). This clade was closest to reference strain *R. oryzae* NRRL18148, suggesting the three *R*. *delemar* isolates were mis-identified by ITS and D1/D2. Clade 8 comprised four *R*. *delemar* linen-associated and regional isolates that were recoved in months 4 and 5. Within each clade, isolates were distinct from each other based on core protein phylogeny, genome size, %GC and/or protein content. Singleton isolates did not cluster with any other study isolates.

## Discussion

This is the first study to perform WGS on epidemiologically linked clinical and environmental *Rhizopus* or Mucorales isolates. Using a comprehensive approach that combined core protein phylogenetic and global genome feature analyses, we were unable to link mucormycosis cases to environmental sources. However, we demonstrated that two SOT recipients with mucormycosis diagnosed in closely situated buildings at our medical centre were infected with *R. microsporus* isolates that were highly similar by core phylogeny and global genome features, and that might have been derived from a common parent strain. A definitive source for these infections is unclear, as a corresponding strain was not recovered from cultures of linen, hospital or linen agency environments, or the surrounding community. Nevertheless, the findings suggest that the patients may have been exposed to a reservoir that was not detected through our surveillance. It is also possible that the responsible strain was distributed widely throughout the greater geographical region, and the two patients were exposed independently to different environmental sources. In some regards, our findings are similar to those of a recent French study, in which two burns patients with mucormycosis were adjudged by WGS to be infected by the same *M. circinelloides* strain, for which a source was not identified [25]. The present study was unique for its inclusion of environmental isolates, and for the striking temporal and spatial distances between patients infected with the highly related isolates (7 months apart, in separate hospitals). Our results attest to the promise and limitations of WGS as a tool for epidemiological investigation of mucormycosis, the complexity of Mucorales genomes, and the environmental burden and genetic diversity of Mucorales.

To date, few studies have employed WGS to investigate possible mucormycosis outbreaks and none of that subset have examined epidemiologically linked environmental strains. In the French study mentioned above, burns patients were infected with strains that clustered within four phylogenomic clades; besides the two patients who shared a common strain (defined by percentage nucleotide differences), patients were infected with genetically diverse isolates. In a study from Edmonton, *Rhizomucor pusillus* that infected a lung transplant and a heart transplant recipient over 6 months were found to be phylogenomically distinct by core genome SNP analysis [26]. A third study included *Apophysomyces trapeziformis* isolates from patients with community-acquired mucormycosis following a tornado in Joplin, MO [24, 27]. Whole genome SNP analysis revealed three phylogenomic clades, each of which comprised at least some strains with ‘identical or nearly identical’ genomes. None of the previous studies included environmental Mucorales isolates. It is unclear if strains defined as identical in the Joplin and French studies would be considered indistinguishable by our approach.

Until recently, putative hospital-acquired mucormycosis cases were investigated by genotyping at ITS, D1/D2 or other conserved loci [21, 22, 45]. ITS and D1/D2 sequencing is the gold standard for species identification in clinical practice, but we showed that these sequences mis-identied several isolates based on WGS data. Moreover, ITS and D1/D2 sequences failed to capture *Rhizopus* strain- and species-level diversity (Fig. S1). In a WGS survey and phylogenomic reconstruction, species-level distinctions within the genus *Rhizopus* also were found to be misleading [23]. Likewise, we demonstrated that phylogenetic analysis of multiple core protein-coding genes led to false conclusions of genetic relatedness between strains, as evident particularly within clades 1, 5 and 6 (Fig. 3). For example, *R. arrhizus* strains L36-GL22 and L41-GL31 (clade 6) were very similar by phylogenomic analysis, but they were clearly separated by genome size. We observed several *R. microsporus* genomes with high phylogenomic similarity across numerous conserved proteins that were quite divergent in global genomic characteristics (Clade 1, Fig. 3). Therefore, a multifaceted strain typing strategy such as ours may be a model for future investigations. SNP analysis was infeasible in our dataset due to low overall sequence similarity across genomes, fragmented DNA assemblies, and limited chromosomal alignments that stemmed from repeat-rich Mucorales genomes. There is a pressing need to standardize WGS-based typing methods for Mucorales and to validate interpretive criteria for strain relatedness. These efforts will require robust, supportive data from re-sequencing a number of identical strains and type strains for the new lineages, with the use of long-read technology to facilitate chromosome-resolved genome assemblies. Such assemblies would allow for SNP profiling of similar strains and act as an enabling dataset for other researchers.

We demonstrated that our hospitals and surrounding communities were home to genomically diverse Mucorales. This observation is consistent with the genetic diversity of *Aspergillus fumigatus* described previously in hospital environments [46, 47]. It is plausible that mucormycosis or mucormycosis outbreaks can be caused by various species and strains, as reported in several investigations, rather than a single clone [24, 25]. We found clear evidence of local genetic relationships among our Mucorales isolates. In general, regional strains were more closely related to each other than to previously sequenced strains from elsewhere in the USA. The genetic relationship between strains also varied temporally, as strains from certain clades were detected throughout the study while others were found at particular time points. Meteorological conditions, construction and other factors change over time, which is likely to impact Mucorales populations and distribution [48]. As shown here, the end result within a hospital or community is a dynamic ecology of new, transient and more entrenched lineages. The implications of our findings for epidemiological investigations are that a failure to demonstrate definitive associations between clinical and environment strains does not preclude hospital-acquired mucormycosis or an outbreak, assessments of hospital-acquired mucormycosis should use WGS on several isolates from an individual patient and a large number of environmental isolates, and investigations are best conducted at the time of active cases, which may be difficult due to delayed recognition of case clusters [20, 25].

We acknowledge that any study such as ours is limited by the impossibility of comprehensively sampling environmental sites for Mucorales within hospitals or a geographical region. The difficulty in linking clinical isolates to environmental sources highlights the importance of detailed, non-molecular epidemiological information in conducting mucormycosis invesigations, and in accurately intepreting WGS data. The data here are insufficient to propose definitions for hospital-acquired versus community-associated mucormycosis. Future WGS studies, if conducted thoughtfully, may increase our understanding of concepts crucial for these definitons, such as incubation period for disease, and persistence of individual strains at sites of colonization in patients or within environmental reservoirs. Likewise, we cannot draw definitive conclusions about the evolution of Mucorales strains *in vivo*. A previous study did not observe genomic changes in an *M. circinelloides* strain that was passed though mice three times by lateral tail vein injection and recovery from infected brains [25]. However, more extensive investigation is needed in this area. Finally, we performed WGS on isolates recovered from cultures that were carefully initiated with a single spore, in order to minimize mixed Mucorales strains that can confound determinations of genomic variations [28].

In conclusion, the prevalance of mucormycosis and recognition of disease outbreaks is likely to increase as numbers of at-risk immunosuppressed patients continue to grow. WGS is poised to alter our understanding of Mucorales biology and mucormycosis epidemiology. As we show here, incomplete analyses of WGS data may lead to spurious conclusions about genome content and strain relatedness, and potentiate the confusion that is already rife regarding Mucorales taxonomy and mucormycosis epidemiology. As WGS data are gathered from multiple studies, it will be possible to generate higher quality genomes, construct chromosome-resolved assemblies, and define precise genus- and species-level distinctions. An important question for the future is whether any Mucorales strain is capable of causing human disease, or if particular strains or clades have attributes that facilitate survival and proliferation in humans and environmental milieu. In the latter scenario, phylogeny or genomic characteristics may provide insight into pathogenesis, and define relative risks posed to patients by Mucorales populations. Finally, regardless of improvements in WGS data and advances in analytical methods, genomic investigations of mucormycosis cases will remain adjuncts to well-conducted, shoe-leather epidemiological studies.

## Supplementary Data

Supplementary material 1
