# Supplementary material for: Genetic diversity of clinical and environmental Mucorales isolates obtained from an investigation of mucormycosis cases among solid organ transplant recipients
Source: Microb Genom. 2020 Nov 27;6(12):mgen000473. doi: 10.1099/mgen.0.000473 (PMC8116672; doi:10.1099/mgen.0.000473)

Supplemental Table 1. Assembly statistics of 72 clinical and environmental isolates and 17 previously sequenced Mucorales strains.

Genome	Contigs (>= 0 bp)	ımber of Conti	LongestContig	GenomeSize	Completeness(%)	GC(%)	N50	#Orfs	#Proteins	n and speciation by ITS and D1/D2	Month recovered
P5-13	13107	10253	260634	4.992E+07	97.6	35.36	43651	18107	18107	*R. arrhizus var. arrhizus*	4.92
R1-11	21257	17179	218359	6.007E+07	96.9	35.27	23843	21771	21771	*R. arrhizus var. arrhizus*	4.89
R2-8	13023	10231	221137	4.872E+07	97.6	35.39	28389	17705	17705	*R. arrhizus var. arrhizus*	1387.05
L1-3	14479	11687	139302	4.865E+07	97.2	35.36	18734	17810	17810	*R. arrhizus var. arrhizus*	1387.08
Rhizopus oryzae NRRL18148	14653	8790	323932	4.53E+07	97.6	35	44395	15786	15786	Reference	Reference
L45-GL21	59808	16482	62422	4.887E+07	95.9	40.07	9552	15741	15741		19.54
L29-GL13	41933	8089	52661	3.618E+07	93.1	35.93	7575	13125	13125	*R. arrhizus* var *delemar*	17.51
L17-GL2	44516	12518	62438	4.321E+07	96.2	36.9	11508	14837	14837	Reference	14.82
L18-GL30	36254	9340	73356	4.077E+07	95.5	35.33	12540	14444	14444	*R. arrhizus var. arrhizus*	1396.98
L13-GL26	34258	9045	67031	3.993E+07	95.9	35.29	11545	14322	14322	*R. arrhizus var. arrhizus*	1396.66
L25-GL8	93200	14631	30470	3.889E+07	92.7	36.45	4686	14471	14471	*R. arrhizus var. arrhizus*	1398.30
L30-GL18	50267	9842	60479	4.080E+07	95.9	35.36	11578	15173	15173	*R. arrhizus var. arrhizus*	17.51
L31-GL17	39558	10311	60373	4.111E+07	95.1	35.94	12000	14560	14560	*R. arrhizus var. arrhizus*	17.51
L14-GL11	38946	9604	62428	4.083E+07	95.5	35.32	12122	14520	14520	*R. arrhizus var. arrhizus*	14.75
L21-GL1	68625	16320	92586	4.907E+07	95.8	40.24	9249	15324	15324	*R. arrhizus var. arrhizus*	15.05
L19-GL10	38362	10005	66776	4.089E+07	95.2	35.4	11275	14511	14511	*R. arrhizus var. arrhizus*	14.82
L2-2	30769	26487	82710	9.272E+07	95.9	35.21	7924	34795	34795	*R. arrhizus* var *delemar*	4.92
Rhizopus oryzae 99-133	4317	3888	381670	4.13E+07	97.3	35.35	58403	14453	14453	Reference	Reference
R3-6	12991	11133	68734	4.811E+07	96.6	35.3	11635	17861	17861	*R. arrhizus* var *delemar*	4.89
R4-5	10338	8126	180755	4.583E+07	97.9	35.34	31393	16401	16401	*R. arrhizus* var *delemar*	4.89
ıizopus delemar NRRL21447 type	1177	1175	583253	3.87E+07	96.9	35.5	143731	12773	12773	Reference	Reference
R5-7	9823	7994	290107	4.534E+07	97.9	35.31	32785	16124	16124	*R. arrhizus* var *delemar*	4.89
L39-16	12784	9718	296030	4.770E+07	98.3	35.46	31036	17441	17441	*R. arrhizus* var *delemar*	4.13
Mucor racemosus CDC-B9738	5266	4963	480951	7.50E+07	98.6	33.26	40443	21264	21264	Reference	Reference
R6-4	4021	3241	154291	2.778E+07	97.6	37.33	33578	11128	11128	*R. microsporus*	4.89
P9-GL28	10841	2288	167324	2.507E+07	97.6	37.19	34008	9149	9149	*R. microsporus*	19.11
L32-GL19	59026	16176	185196	3.635E+07	95.9	41.61	17031	15693	15693	*R. microsporus*	17.51
P3-GL61	6133	2074	259386	2.490E+07	97.3	37.17	36268	9136	9136	*R. microsporus*	3.02
P6-GL35	6229	2057	243671	2.494E+07	97.5	37.18	37278	9158	9158	*R. microsporus*	10.89
P6-GL58	7240	2139	168971	2.501E+07	97.2	37.17	38009	9144	9144	*R. microsporus*	10.89
Rhizopus microsporus M201021	3431	3373	187629	4.57E+07	92.8	36.92	29135	17767	17767	Reference	Reference
R7-12	15299	12661	234433	7.591E+07	97.6	36.02	34960	28423	28423	*R. arrhizus var. arrhizus*	4.89
aksenaea vasiformus CDC-B407	2417	2149	560921	4.24E+07	96.9	42.66	79983	14461	14461	Reference	Reference
ksenaea oblongisporus CDC-B33	1702	1489	549295	4.07E+07	96.5	41.91	109559	13124	13124		Reference
ephalastrum racemosum CDC-B	1035	942	1285691	2.95E+07	96.6	47.18	332696	11055	11055		Reference
ıcephalastrum monosporum B89	1284	1133	801691	2.95E+07	96.9	48.35	221465	11355	11355		Reference
Mortierella alpina CDC-B6842	1183	1034	745939	3.95E+07	96.2	50.41	144249	14686	14686		Reference
Jmbelopsis isabellina CDC-B731	58	47	1877206	2.19E+07	98.2	41.98	1154400	8484	8484		Reference

Supplemental Fig. 1. Phylogenetic relationships among 72 *Rhizopus* isolates by ITS (A) and D1/D2 (B) sequencing. Bootstrap values are for 100 iterations.

A. ITS phylogeny.

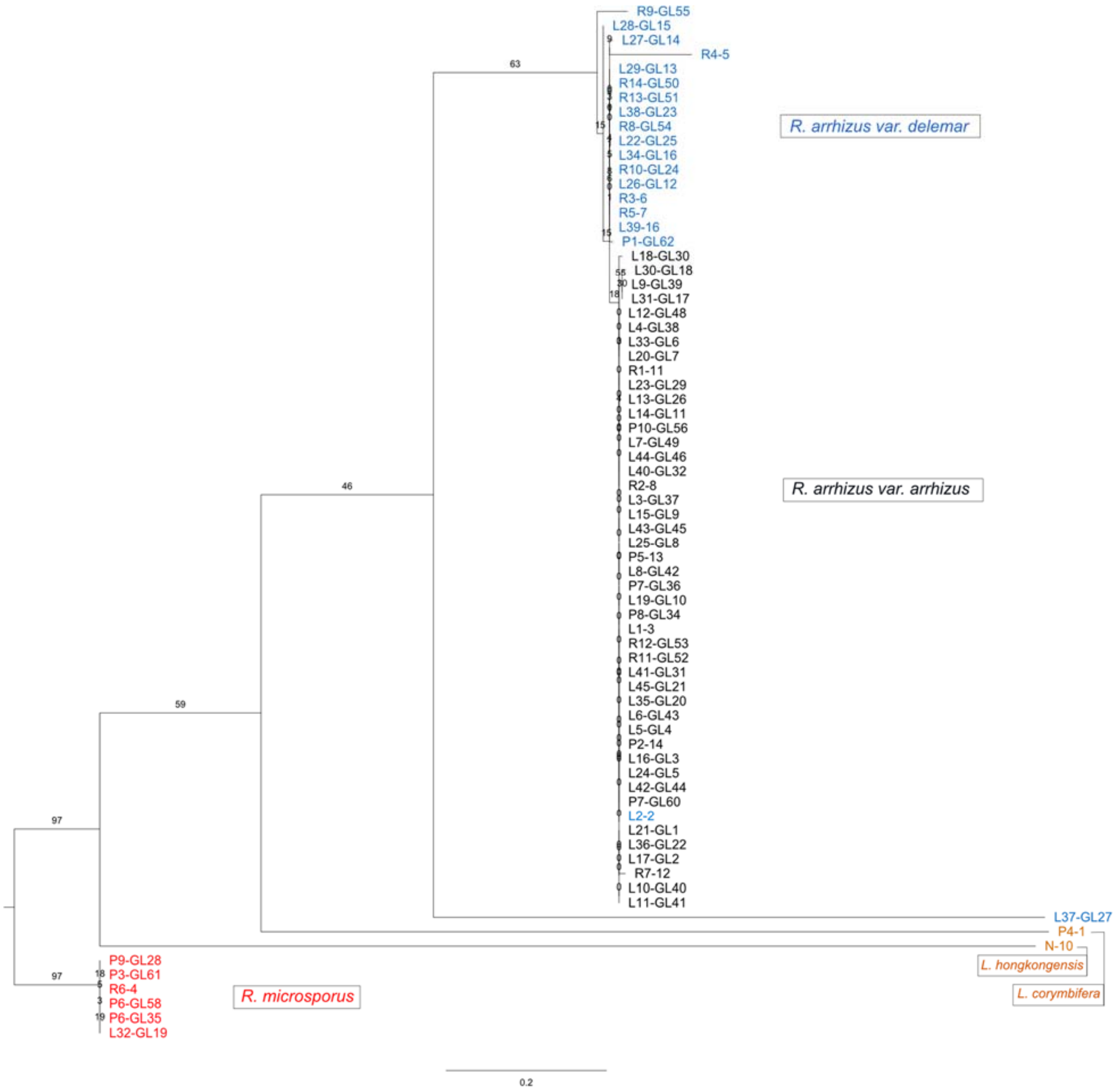

B. D1/D2 phylogeny.

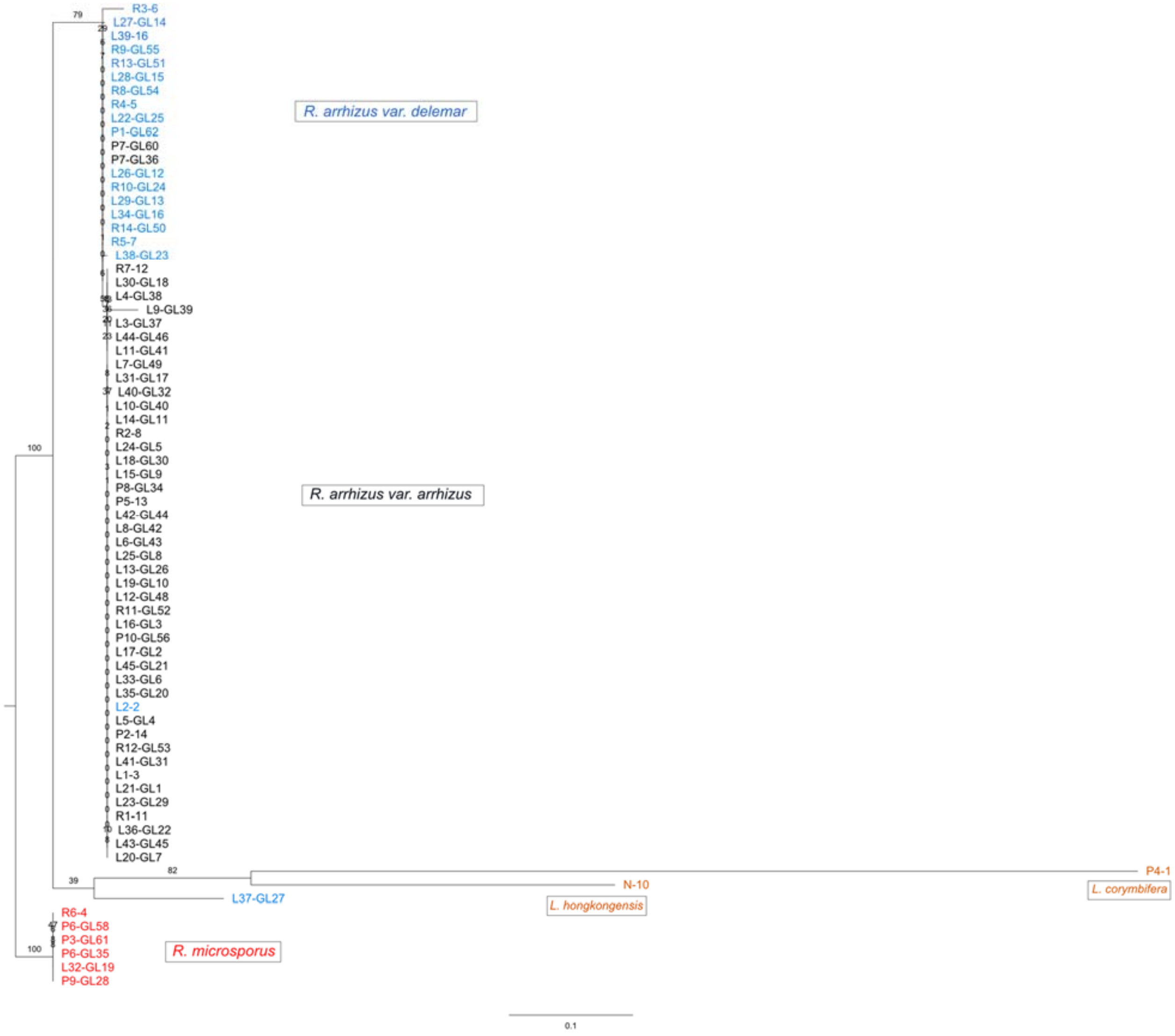

Supplement: Supplementary material 1 [file mgen-6-473-s001.pdf]
